# Histone H3 lysine 4 methyltransferases and demethylases in self-renewal and differentiation of stem cells

**DOI:** 10.1186/2045-3701-3-39

**Published:** 2013-10-09

**Authors:** Bingnan Gu, Min Gyu Lee

**Affiliations:** 1 Department of Molecular and Cellular Oncology, The University of Texas MD Anderson Cancer Center, 1515 Holcombe Blvd., Houston, TX 77030, USA

**Keywords:** Histone methylation, H3K4, Methyltransferase, Demethylase, Stem cell, Self-renewal, Differentiation

## Abstract

Epigenetic mechanisms are fundamental to understanding the regulatory networks of gene expression that govern stem cell maintenance and differentiation. Methylated histone H3 lysine 4 (H3K4) has emerged as a key epigenetic signal for gene transcription; it is dynamically modulated by several specific H3K4 methyltransferases and demethylases. Recent studies have described new epigenetic mechanisms by which H3K4 methylation modifiers control self-renewal and lineage commitments of stem cells. Such advances in stem cell biology would have a high impact on the research fields of cancer stem cell and regenerative medicine. In this review, we discuss the recent progress in understanding the roles of H3K4 methylation modifiers in regulating embryonic and adult stem cells’ fates.

## Introduction

Stem cells have long-term self-renewing activity and can commit to multiple cell types upon differentiation signals. Since Yamanaka and colleagues demonstrated that the four DNA-binding transcription factors Oct4, Sox2, c-Myc, and Klf4 transform fibroblasts into a type of pluripotent cells known as induced pluripotent stem cells, the importance of transcription factors in cellular reprogramming has been more recognized [1]. However, because the reprogramming efficiency of these four factors is low, it is evident that additional layers of co-regulatory mechanisms exist besides transcription factor-driven regulation [2]. In fact, a recent study demonstrated that the histone modification and DNA methylation profiles differ in one-third of the genome between human embryonic stem (ES) cells and primary fibroblasts [3], indicating that such remarkable epigenetic difference may serve as a major molecular mechanism in determining cellular characteristics of these two cell types. Notably, the functions of epigenetic modifiers in stem cell fate decision have been intensively studied.

Histone lysine methylation has been widely accepted as a key epigenetic modification. Unlike acetylation, the methylation does not change the charge of lysine residues and thus has a minimal direct effect on DNA-histone association. Rather, the different methylation status of specific histone lysines can serve as a unique platform for recruiting methylation “reader” proteins that activate or repress genes’ transcriptional activity. In general, histone H3 lysine 4 (H3K4), H3K36, and H3K79 methylation are gene activation marks, whereas H3K9, H3K27, and H4K20 methylation are gene-repressive modifications [4].

Histone lysine methylation is generated by a battery of histone methyltransferases (HMTs) that transfer the methyl group from S-adenosylmethionine to specific lysine residues. For example, H3K4 methylation is mediated by several SET [Su(var)3-9, Enhancer of zeste, Trithorax] domain-containing methyltransferases, including mixed lineage leukemia 1–5 (MLL1−5), SET1A/B, SET7/9, SET and MYND domain-containing protein 1–3 (SMYD1−3), Absent, Small, or Homeotic 1-like (ASH1L), SET domain and Mariner transposase fusion gene (SETMAR), and PR domain zinc finger protein 9 (PRDM9) [5-24]. Methylated lysines exist in three forms: mono-, di- and tri-methylation (me1, me2, and me3).

Similar to other histone modifications, histone methylation can be reversed by histone demethylases (HDMs). The first identified lysine-specific demethylase 1 [LSD1; also known as FAD-binding protein BRAF35-HDAC complex, 110 kDa subunit (BHC110) and Lysine-specific demethylase 1A (KDM1A)], together with LSD2, belongs to the polyamine oxidase family. LSD1 and LSD2 remove methyl groups from di- and monomethylated H3K4 but are unable to demethylate trimethylated H3K4 [25-28]. LSD1 was reported to also have H3K9 demethylation activity [29]. Subsequently, many Jumonji (JmjC) domain-containing histone demethylases have been discovered. In particular, the JARID1 family of histone demethylases (JARID1A−D) can erase H3K4me3 and H3K4me2 [30-35].

In this review, we summarize the recent progress in understanding the functions of H3K4 methyltransferases and demethylases in modulating stem cells’ fates.

### H3K4 methylation

**Figure 1:**
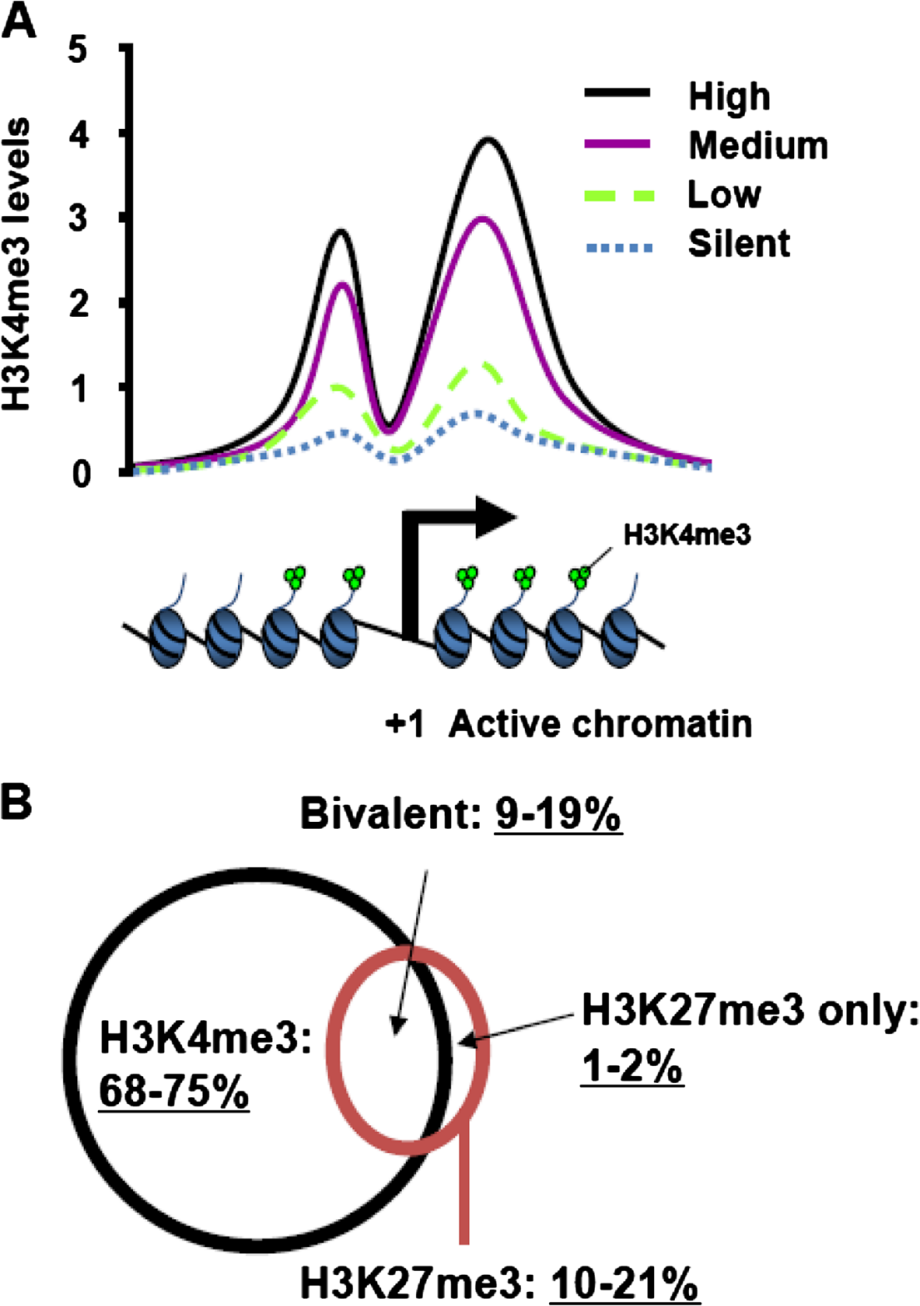
**H3K4me3 marks actively transcribed and poised gene promoters in mammals. (A)** The genome-wide correlation of mRNA expression levels (High, Medium, Low, and Silent) with H3K4me3 levels at human gene promoters. Note that a dip of H3K4me3 levels may be associated with the nucleosome-free region around the transcriptional start site (TSS). Adapted from [39]. **(B)** The Venn diagram showing the percentage of genes that have H3K4me3 and/or H3K27me3 in their promoters in mouse and human ES cells. All percentages are based on about total 18,000 genes. The “bivalent” denotes the promoters that contain both H3K4me3 and H3K27me3 marks. Adapted from [36,37,43].

H3K4me3 occupies as many as 75% of all human gene promoters in several cell types (e.g., ES cells), indicating that it plays a critical role in mammalian gene expression [36,37]. In fact, H3K4me3 is required to induce critical developmental genes in animals, including *Drosophila* and several mammals, and is important for animal embryonic development [38]. H3K4me3 levels are positively correlated with gene expression levels [39,40] (Figure 1A).

Although H3K4me3 is clearly associated with actively transcribed genes, however, studies have demonstrated that H3K4me3 is localized around the transcription initiation sites of numerous unexpressed genes in human ES cells, primary hepatocytes, and several other cell types [36,37,41]. In particular, it frequently co-resides with the repressive mark H3K27me3 in the promoters of critical differentiation-specific genes [e.g., *Homeobox* (*HOX*) gene clusters] that are transcriptionally inactive in ES cells [36,37,42,43] (Figure 1B). It has been proposed that the “bivalent” domains, composed of H3K4me3 and H3K27me3, may maintain differentiation-specific gene promoters in a repressive status in self-renewing stem cells but be poised for prompt gene activation upon differentiation stimuli [42]. Consistent with this, many bivalent genes have increased H3K4me3 levels and decreased H3K27me3 levels while being transcriptionally activated during differentiation. Interestingly, recent studies demonstrated that most bivalent domains are occupied by LSD1 [44,45], indicating that it plays a role in maintaining low levels of dimethylated H3K4 (H3K4me2) that are often co-localized with H3K4me3. For these reasons, H3K4me3 is classified as a chromatin landmark for transcriptionally active or poised genes in ES cells [41].

Compared with mouse thymocytes, mouse ES cells contain higher levels of total genomic H3K4me3 and have higher H3K4me3 occupancy at the promoter of the pluripotent gene *Oct4*[46]. In agreement with this, global decreases in H3K4me3 levels occur during retinoic acid (RA)-induced differentiation of mouse ES cells [47]. In addition, there are dynamic changes in H3K4me3 profiles at specific sets of genes during ES cell differentiation. Such global and local changes in H3K4me3 profiles are partly because levels of H3K4me3-regulatory factors [e.g., WD repeat-containing protein 5 (WDR5), MLL1 and MLL3] are modulated [47]. It is believed that higher H3K4me3 levels allow the ES cell genome to be more open and transcriptionally permissive by recruiting chromatin-modifying factors. Therefore, unique H3K4me3 profiles at pluripotent and differentiation-specific genes may be key determinants of cellular identity.

Most H3K4me3-containing promoters are also occupied by H3K9/H3K14 acetylation [41]. In transcriptionally active genes, H3K36me3 and H3K79me2 are significantly enriched downstream of H3K4me3-containing promoters: H3K36me3 peaks toward the 3′ end of genes in gene bodies, whereas H3K79me2 is located toward the 5′ end [41]. Therefore, H3K4me3 likely cooperates with other histone marks for gene activation. The combinatorial arrangement of H3K4me3 and other histone marks may support, at least in part, the “histone code” hypothesis [48].

H3K4me2 decorates genomic regions independently of H3K4me3, although most of it overlaps with H3K4me3 near the transcription start sites [49]. H3K4me2 may have an antagonistic effect on DNA methylation [50]. Monomethylated H3K4 (H3K4me1) also co-occupies regions near the start sites with H3K4me3. Apart from the transcription start sites, H3K4me1, together with H3K27 acetylation, specifies enhancer regions [51,52]. In summary, H3K4me1, H3K4me2 and H3K4me3 have a commonality for gene activation, although their subsets play distinct roles in modulating chromatin function.

### H3K4 methyltransferases

Some H3K4 methyltransferases are well conserved in different species. In yeast, the Set1 complex, also called Complex of Proteins Associated with Set1 (COMPASS), catalyzes the mono-, di- and trimethylation of H3K4 [5,8]. The protein complex is composed of the catalytic component of Set1 and seven other regulatory subunits (Cps60, Cps50, Cps40, Cps35, Cps30, Cps25, and Cps15) that are essential for full enzyme activity [38] (Table 1). In *Drosophila*, there are three Set1 homologs: dSet1, Trithorax (Trx), and Trithorax-related (Trr). The deletion of any of their genes results in lethality in flies, indicating that their target genes may not be redundant. In particular, loss of *dSet1*, but not *Trx* or *Trr*, leads to a global reduction of H3K4me2/3, suggesting that *Trx* and *Trr* have more specialized functions [38]. Human SET1A, SET1B, and MLL1−4 are yeast Set1 homologs and are related to dSet1 (the counterpart of SET1A and SET1B), Trx (the counterpart of MLL1 and MLL2), and Trr (the counterpart of MLL3 and MLL4) in *Drosophila*. Other SET domain-containing histone methyltransferases that methylate H3K4 but are not closely related to yeast Set1/COMPASS have also been identified and include MLL5, SET7 (also called SET9), SMYD1-3, SETMAR, and PRDM9 [6,15,24].

**Table 1:** Subunit composition of H3K4 methyltransferase complexes in yeast and human

**Yeast SET1**	**Human SET1A**	**Human SET1B**	**Human MLL1**	**Human MLL2**	**Human MLL3**	**Human MLL4**	Human MLL5*
SET1	SET1A	SET1B	MLL1	MLL2	MLL3	MLL4	Mll5
Cps60/Bre2	ASH2L	ASH2L	ASH2L	ASH2L	ASH2L	ASH2L	HCF1
Cps50/Swd1	RBBP5	RBBP5	RBBP5	RBBP5	RBBP5	RBBP5	OGT
Cps30/Swd3	WDR5	WDR5	WDR5	WDR5	WDR5	WDR5	STK38
Cpd25/Sdc1	DPY-30	DPY-30	DPY-30	DPY-30	DPY-30	DPY-30	PPP1CA
Cps40/Spp1	CFP1	CFP1					PPP1CB
Cps35/Swd2	WDR82	WDR82					PPP1CC
Cps15/Shg1		BOD1/BOD1L					ACTB
	HCF1/2	HCF1/2	HCF1/2	HCF1/2	NCOA6	NCOA6	
			MENIN	MENIN	UTX	UTX	
				PSIP1	PTIP	PTIP	
					PA1	PA1	

* The subunits of MLL5 are not related to those of SET1, SET1A/B, and MLL1−4.

SET1A/1B and MLL1−4 are present in multi-protein complexes and share common core subunits, such as WDR5, Retinoblastoma-binding protein 5 (RBBP5), ASH2L, and Dumpy-30 (DPY-30), which are also highly conserved in yeast and flies [38] (Table 1). Several studies have demonstrated that these core subunits are indispensable for the enzyme activity of methyltransferases and biological functions [53-55]. In addition to common core subunits, there are unique subunits in the individual H3K4 methyltransferase complexes: WDR82 and CXXC finger protein 1 (CFP1) in the SET1 complex; Multiple endocrine neoplasia type 1 (MENIN) and PC4 and SFRS1-interacting protein 1 (PSIP1) in MLL1 and 2 complex; Host cell factor 1/2 (HCF1/2) in SET1, MLL1, and MLL2 complexes; and PAX transcription activation domain interacting protein 1 (PTIP), PTIP-associated protein 1 (PA1), Nuclear receptor coactivator 6 (NCOA6), and Ubiquitously transcribed X chromosome tetratricopeptide repeat protein (UTX) in the MLL3 and MLL4 complexes [12,16,19,22,56-63] (Table 1). These subunits may play important roles in recruiting H3K4 methyltransferases to specific genes and integrating additional histone-modifying capacities (see below).

#### MLL1 and MLL2

**Figure 2:**
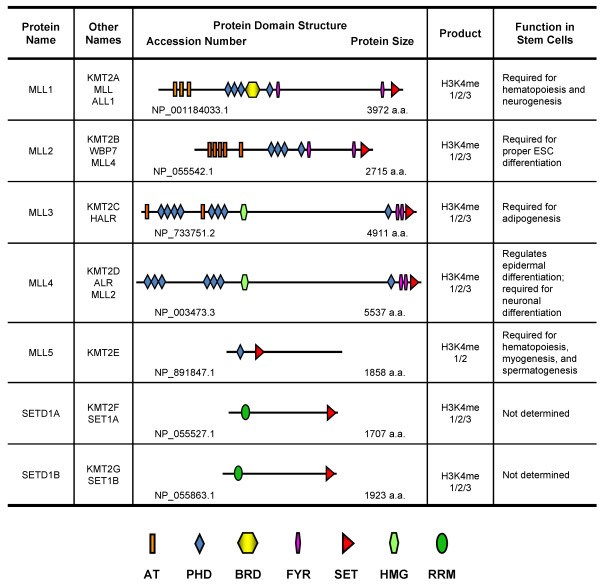
**Protein domain architectures and stem cell function of MLL/SET1 H3K4 methyltransferases.** AT: AT-hook DNA binding domain; PHD: Plant Homeo Domain; BRD: Bromodomain; FYR: FY-rich domain; SET: Su(var)3-9, Enhancer of zeste, Trithorax domain; HMG: High Mobility Group domain; RRM: RNA Recognition Motif.

*MLL1* (also known as *MLL* and *KMT2A*) was initially cloned from acute myeloid and lymphoid leukemia that contain frequent *MLL1* chromosomal fusions and translocations [64-66]. The *MLL1* gene encodes a protein of 3,972 amino acids; this protein contains several highly conserved functional domains, including the N-terminal AT-hook DNA binding domains, Plant homeo domains (PHD), a Bromo domain, and the catalytic SET domain (Figure 2). Inside cells, MLL1 protein is cleaved into MLL-N (320 kDa) and MLL-C (180 kDa) by Taspase I; these two large fragments dimerize through FY-rich motifs to form the functional MLL complex *in vivo*[67,68].

Homozygous deletion of *Mll1* is embryonic lethal; *Mll1*^+/−^ mice display retarded growth and hematopoietic defects [69,70]. Specifically, expression of the key developmental genes, including *Hoxa7* and *Hoxc9*, were shifted from the anterior boundaries toward the posterior regions in *Mll1*^+/−^ embryos and were lost in *Mll1*^−/−^ mice [69]. In addition, recent studies using a tissue-specific knockout mouse model revealed that Mll1 is essential for sustaining adult hematopoiesis [71,72]. *Mll1* is not required for survival, proliferation, and differentiation of subventricular zone neural stem cells but plays an essential role in neurogenesis in the postnatal mouse brain [73]. Mechanistically, Mll1 directly occupies the promoter of *Distal-less homeobox 2* (*Dlx2*), a critical regulator of neurogenesis, and is required to resolve the poised bivalent state to the actively transcribed status with predominant H3K4me3 during neurogenesis of neural stem cells [73].

MLL2 (also called MLL4 and KMT2B) has a similar protein domain structure to that of MLL1 and was found to be the MLL1 paralog [74]. Like Mll1, Mll2 is widely expressed during development and in adult tissues. *Mll2-*null mice die before embryonic day E11.5, with drastically reduced expression of *Hoxb2* and *Hoxb5*[75]. However, *Mll2* may be only required briefly for development, because it appears to be dispensable for mouse development after E11.5 [76]. *Mll2*^−/−^ ES cells maintain pluripotency, have increased apoptotic activity, and undergo skewed cellular differentiation along three germ layers [77]. Therefore, Mll1 and Mll2 are unlikely redundant for gene regulation during early embryonic development. In support with this notion, the phenotypes of *Mll1* and *Mll2* knockout mice are different in adult tissues. For example, hematopoietic-specific loss of *Mll1* showed defects in hematopoiesis [71,72], whereas *Mll2* loss did not show any aberrant blood profiles and notable pathology [76].

#### MLL3 and MLL4

MLL3 (also called HALR/KMT2C) and MLL4 (alias ALR/KMT2D) are mammalian counterparts of *Drosophila* Trr and were co-purified as transcriptional coactivator complexes [14,78-80]. MLL3 and MLL4 associate with nuclear hormone receptors in both *Drosophila* and mammals. For example, the MLL3/MLL4 complex is recruited to *HOXC6* gene and activates its transcription in an estrogen receptor-dependent manner [79]. Frequent somatic loss-of-function mutations have been identified in *MLL3* and *MLL4* genes in human cancers, including colorectal cancer, non-Hodgkin B-cell lymphoma, and medulloblastoma [81-85]. Consistently, a recent study reported that *trr* gene product suppresses cell growth in *Drosophila* eye imaginal discs. Of interest, *trr* mutation markedly reduced H3K4 monomethylation levels without significantly changing H3K4 di- and trimethylation levels [86], in agreement with earlier findings that Trr is a major H3K4 mono-methyltransferase for *Drosophila* enhancers [87]. *Mll3* homozygous mutant mice, which have an in-frame deletion of a 61-aa catalytic core of the SET domain, exhibited reduced white adipose tissue, stunted growth, and slow cellular doubling rate [88,89]. During epidermal differentiation, the MLL4 complex is recruited to differentiation-related genes via the transcription factor GRHL3/GET1 and collaboratively activates the epidermal progenitor differentiation program [90].

Recently, we found that MLL4 is essential for the neuronal differentiation of human NT2/D1 stem cells [91]. Mechanistically, the neuron-specific gene *NESTIN* and key developmental genes *HOXA1–3* are activated by MLL4 during RA-induced differentiation. Intriguingly, the tandem PHD_4-6_ of seven PHD motifs in MLL4 (Figure 2) specifically recognized unmethylated or asymmetrically dimethylated histone H4 Arg 3 (H4R3me0 or H4R3me2a) and is required for MLL4′s nucleosomal methyltransferase activity and MLL4-mediated differentiation. H4R3 symmetric dimethylation (H4R3me2s), a gene-repressive mark, blocks the binding activity of MLL4′s PHD_4-6_. Consistent with this, knockdown of the protein arginine methyltransferase 7, which is involved in generation of H4R3me2s, increases MLL4 occupancy and H3K4me3 levels at the MLL4 target gene promoters and enhances the MLL4-dependent neural differentiation program. Therefore, these results revealed that the trans-tail regulation of MLL4-catalyzed H3K4me3 by protein arginine methyltransferase 7-controlled H4R3me2s serves as a novel epigenetic mechanism underlying neuronal differentiation of human stem cells.

#### MLL5

Independent studies have demonstrated that MLL5 is required for hematopoiesis [92-94]. Moreover, MLL5 promotes myogenic differentiation by controlling expression of cell cycle genes (e.g., *Cyclin A2*) and myogentic regulator genes (e.g., *Myogenin*) [95]. *Mll5* knockout male mice are sterile, at least in part because of deregulated expression of genes that are required for terminal differentiation during spermatogenesis [96]. Of interest, although MLL5 was reported to be inactive [92,95], GlcNAcylation of MLL5 greatly increased MLL5′s enzymatic activity towards H3K4me1/2 and facilitated RA-induced granulopoiesis in human HL60 promyelocytes [24].

#### SET1A and SET1B

Human SET1A and SET1B have an N-terminal RNA recognition motif and a C-terminal enzymatic SET domain (Figure 2). The SET1A complex was purified as a multi-protein complex that associates with CFP1 [19]. CFP1 is required for stem cell differentiation and interacts with unmethylated CpGs via its zinc finger domain CXXC [97]. Interestingly, *Cfp1*^−/−^ ES cells displayed aberrant H3K4me3 peaks at numerous ectopic sites (i.e., distinct regions outside annotated CpG islands), suggesting that CFP1 recruits the SET1 complex to CpG island-containing promoters and consequently prevents it from generating H3K4me3 to inappropriate chromatin locations [19,98,99].

A protein sequence analysis revealed that SET1A shares 39% identity with a SET domain protein named SET1B [22]. Although both proteins associate with a similar set of non-catalytic subunits, a confocal microscopy analysis revealed that SET1A and SET1B exhibit distinct subnuclear localizations in euchromatin regions; thus, this suggests that each protein regulates a unique group of target genes [22].

#### ASH1L

ASH1L (also called Ash1) is the human homolog of Ash1, a *Drosophila* Trithorax group protein that is essential for expression of several *HOX* genes. Some reports have indicated that ASH1L primarily acts as a H3K4 methyltransferase [13,100,101], whereas others have reported that human ASH1L specifically mono- and dimethylates H3K36 [102-104]. ASH1L cooperates with MLL1 in *HOX* gene activation and is required for the myelomonocytic lineage differentiation of hematopoietic stem cells [105]. Of interest, a mutation of the SET domain of ASH1L did not decrease *HOX* gene expression, suggesting that ASH1L’s catalytic activity is dispensable for hematopoietic stem cell differentiation [105].

#### SET7/9

SET7 (or called SET9) is an H3K4 mono- and di-methytransferase [6,106-108]. SET7 expression is upregulated during myoblast differentiation [109]. Specifically, SET7 interacts with Myoblast determination protein 1 (MyoD), a central transcriptional factor for myogenic gene expression, and is indispensable for MyoD-mediated muscle differentiation. Knockdown of SET7 impaired the association of MyoD with the promoter and enhancer regions of the myogenic genes (e.g., *Myogenin*) and reduced gene expression by decreasing H3K4me1 levels at its target genes. Intriguingly, SET7 antagonizes Suv39h1-mediated H3-K9 methylation at the myogenic differentiation gene promoters [109].

#### SMYD1−3

Smyd1 (also called Bop) is essential for mouse cardiac differentiation [110]. Consistently, knockdown of Smyd1 in zebrafish embryos results in defective skeletal and cardiac muscle differentiation; this cannot be rescued by the *Smyd1* catalytic mutant, which lacks H3K4 methyltransferase activity [21]. SMYD2 methylates H3K4 and H3K36, as well as tumor-suppressor proteins such as p53 and Retinoblastoma protein (pRB) [23,111-113]. Specifically, SMYD2-mediated monomethylation of p53 K370 attenuates the interaction of p53 with p53 target promoters and consequently antagonizes p53-dependent transcriptional regulation [112]. Unlike SMYD1, cardiac-specific knockout of *Smyd2* has no phenotype during mouse heart development [114]. SMYD3 is a methyltransferase for both H3K4 and H4K5 [15,115]. It is overexpressed in colorectal and hepatocellular cancers and promotes cell proliferation [15]. During zebrafish embryogenesis, SMYD3 appears to be important for cardiac and skeletal muscle development [116].

#### SETMAR

**Figure 3:**
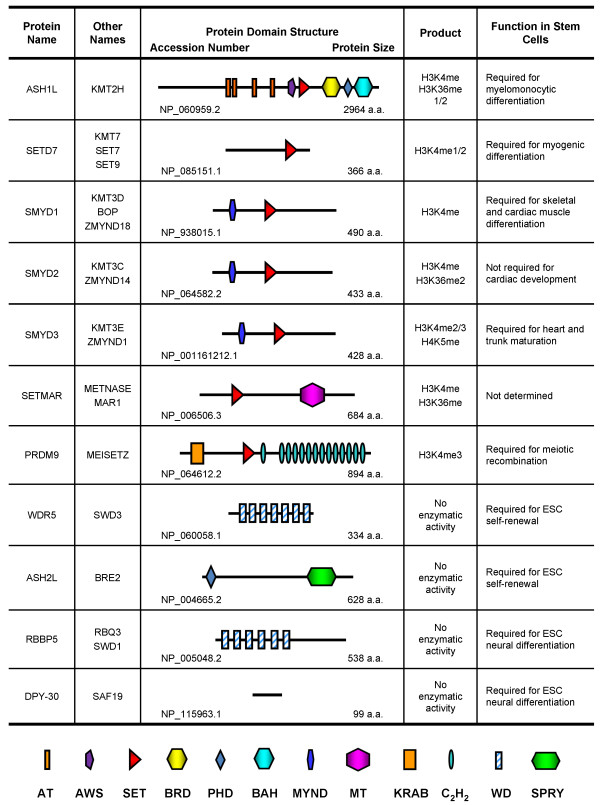
**Protein domain architectures and stem cell function of other H3K4 methyltransferases and core subunits.** AT: AT-hook DNA binding domain; AWS: Associated With SET domain; SET: Su(var)3-9, Enhancer of zeste, Trithorax domain; BRD: Bromodomain; PHD: Plant Homeo Domain; BAH: Bromo Adjacent Homology domain; MYND: Myeloid, Nervy, and DEAF-1 domain; MT: Mariner Transposase domain; KRAB: Krüppel Associated Box domain; C_2_H_2_: C_2_H_2_-type zinc finger; WD: WD40 repeat; SPRY: SplA and Ryanodine domain.

*SETMAR* (also called *METNASE*) encodes a chimeric protein that contains an N-terminal SET domain and a C-terminal mariner transposase domain [117] (Figure 3). The function of SETMAR in stem cells remains unknown. However, SETMAR-catalyzed methylation of H3K4 and H3K36 may lead to an open chromatin structure, which may facilitate its transposase-dependent processes, such as foreign DNA integration and DNA double-strand break repair [20].

#### PRDM9

PRDM9 (also called MEISETZ) is a PR/SET domain-dependent histone methyltransferase that is required for meiotic prophase progression [18]. Deletion of the *Prdm9* gene attenuates H3K4me3 levels, resulting in defective chromosome pairing, impaired sex body formation, damaged meiotic progression, and sterility in both sexes of mice [18]. Mechanistically, Prdm9 binds to 13-base pair DNA elements via its C2H2 zinc fingers. During early meiosis, this binding event may link Prdm9-catalyzed H3K4me3 to mammalian meiotic recombination hotspots that contain the 13-nucleotide DNA elements [118-120].

### Subunits of H3K4 methyltransferases

WDR5, a core subunit of the SET1 and MLL1−4 complexes, plays an important role in ES cell self-renewal and somatic cell reprogramming [47]. WDR5 is highly expressed in ES cells and downregulated upon differentiation. Knockdown of WDR5 resulted in loss of ES cell self-renewal and decreased the generation of induced pluripotent stem cells [47]. WDR5 interacts with OCT4 and activates transcription of the self-renewal factors, such as OCT4 and NANOG, in ES cells. Moreover, WDR5, together with OCT4, NANOG and SOX2, regulates the self-renewal-regulatory network [47]. Similarly, ASH2L is required for the pluripotency of mouse ES cells. ASH2L knockdown resulted in elevated expression of mesodermal lineage differentiation genes [121].

DPY-30 and RBBP5 are other core components of the SET1/MLL methyltransferases. In contrast to ASH2L and WDR5, DPY-30 and RBBP5 were not required for ES cell self-renewal [53]. DPY-30 or RBBP5 knockdown reduces global and neuronal gene-specific H3K4me3 levels, resulting in inefficient RA-induced neural differentiation of mouse ES cells.

Differing biological outcomes for ASH2L and WDR5 from DPY-30 and RBBP5 are surprising because these four proteins are core components of the same SET1/MLL1−4 methyltransferases. These unexpected findings might be explained by the following possibilities. Besides the known SET1/MLL1−4 complexes, some of these subunits may be present in other complexes in the same cells so that they may exert different biological functions from SET1/MLL1−4 complexes. In fact, gel filtration analysis of ES cell nuclear extracts showed that elution profiles of WDR5/OCT4 did not overlap with those of WDR5/ASH2L/RBBP5, suggesting that WDR5 also belongs to another new complex containing OCT4 [47]. Another possible scenario is that cellular levels of some core subunits and H3K4 methyltransferases may be dynamically changed between ES cells and differentiated cells. Such changes might allow certain H3K4 methyltransferase complexes to be dominant over the others or lead to formation of new functional complexes, subsequently affecting expression of stemness genes and differentiation-specific genes. In support with this, during ES cell differentiation, ASH2L and WDR5 levels are down-regulated whereas MLL1 and MLL3 are up-regulated [47,121]. In addition, some H3K4 methyltransferase complexes may have non-redundant cellular function by regulating their unique target genes in a cell type-specific manner, as mentioned earlier. Future studies are required to further understand the distinct roles of the SET1/MLL complexes.

### H3K4 demethylases

The reversibility of histone methylation was not clear until the discovery of the first histone demethylase LSD1 in 2004 [25]. Subsequently, a new class of JmjC-domain-containing proteins was identified that can demethylate methylated lysine residues in histones. The F-box and leucine-rich repeat protein (FBXL11, also known as KDM2A) is the first identified JmjC domain-containing demethylase that removes methyl groups from H3K36me2/1 [122]. The catalytic JmjC domain requires iron and α-ketoglutarate as cofactors to hydroxylate methyl groups [123]. Among this class of demethylases, JARID1A−D (or KDM5A−D) proteins specifically remove the methyl group from H3K4me2/3. NO66, a bifunctional lysine-specific demethylase and histidyl-hydroxylase, can demethylate H3K4me/ H3K36me and hydroxylate a histidyl group of the non-histone protein Rpl8 [124,125]. Not surprisingly, the LSD family (LSD1 and LSD2) and JARID1 family of H3K4 demethylases play important roles in gene transcription in stem cell homeostasis.

#### LSD1 and LSD2

**Figure 4:**
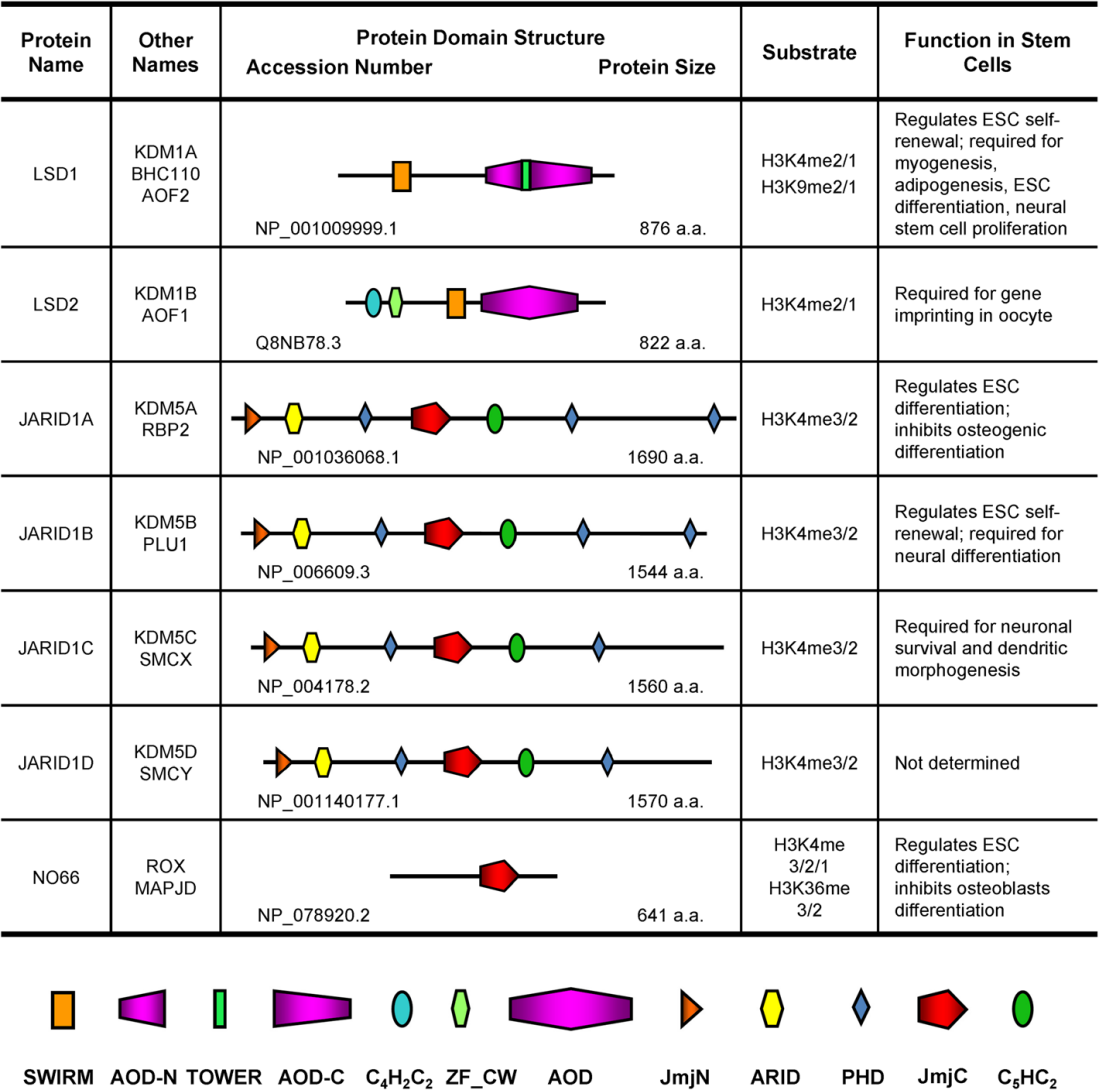
**Protein domain architectures and stem cell function of H3K4 demethylases.** SWIRM: SWI3, RSC8 and MOIRA domain; AOD-N: Amine Oxidase Domain-N terminal; TOWER: LSD1 tower domain; AOD-C: Amine Oxidase Domain-C terminal; C_4_H_2_C_2_: C_4_H_2_C_2_-type zinc finger; ZF_CW: CW-type zinc finger; AOD: Amine Oxidase Domain; JmjN: Jumonji N domain; ARID: AT-rich interactive domain; PHD: Plant Homeo Domain; JmjC: Jumonji C domain; C_5_HC_2_: C_5_HC_2_-type zinc finger.

LSD1 protein contains an N-terminal SWIRM domain and a long C-terminal FAD-dependent amine oxidase domain (AOD). The AOD is divided by an insertion known as the tower domain (Figure 4). LSD1 alone demethylates H3K4me2/1 on histones but not nucleosomes, while the association of Co-REST with LSD1 allows LSD1 to demethylate nucleosomal H3K4 [26,27,126].

Numerous studies in ES cells and neural stem cells strongly suggest that LSD1 is a key histone methylation modifier in transcriptional regulation for stem cell fate determination. *Lsd1*-null mice are embryonic lethal around E6.5, and *Lsd1*-deficient mouse ES cells demonstrate increased cell death and impaired differentiation, such as embryoid body formation defects [127-129]. Similar to mouse ES cells, LSD1 is required for neural stem cell proliferation; it is recruited by the nuclear receptor TLX to repress negative cell cycle regulators, including *p21*, in neural stem cells [130]. Interestingly, LSD1 is indispensable for differentiation of several cell types, including skeletal muscles and adipocytes [131,132]. In mouse ES cells, LSD1 demethylates and stabilizes DNA methyltransferase 1 (DNMT1), and *Lsd1* deletion results in progressive loss of DNA methylation [128]. Moreover, LSD1 and its associated nucleosome remodeling and histone deacetylase (NuRD) complex are recruited to Oct4-occupied enhancers at active stemness genes in ES cells, but the repression activities of LSD1-NuRD may be antagonized by histone acetyltransferases (e.g., p300). During mouse ES cell differentiation, Oct4 and acetyltransferase levels are down-regulated, and LSD1-NuRD decommissions active enhancers by removing H3K4me1 while promoting cellular differentiation [45]. In contrast to the above stem cell studies, seemingly conflicting results regarding the role of LSD1 in ES cells have been reported. Knockdown of LSD1 induces differentiation in human ES cells, which is correlated with de-repression of developmental genes with elevated H3K4me2/3 levels [44]. In addition, *Lsd1*^−/−^ ES cells had a strong potential to generate extraembryonic tissues from the embryoid body [133].

LSD2 (AOF1 or KDM1B) was recently identified as a homolog of LSD1; it demethylates H3K4me2/1 like LSD1 [28,134-136]. Interestingly, unlike LSD1, LSD2 has no tower domain in the AOD region, but contains unique N-terminal zinc fingers, including C_4_H_2_C_2_ and CW-type zinc fingers, which are required for demethylase activity [136,137] (Figure 4). A genome-wide mapping analysis revealed that LSD2 primarily resides in the intragenic regions of actively expressed genes [28]. LSD2 may activate its target genes, possibly via its association with transcriptional elongation factors [28]. *Lsd2* is not essential for mouse development. However, the DNA methylation of several imprinted genes is lost in oocytes from *lsd2*-deleted females [135]. Consequently, the embryos derived from these oocytes exhibited biallelic expression or silencing (i.e., loss of monoallelic expression) of the affected imprinted genes and died before mid-gestation [135]. The molecular mechanism underlying the functional link between H3K4 demethylation and DNA methylation for expression of imprinted genes remains to be investigated.

#### JARID1A

JARID1A (RBP2 or KDM5A) was identified as a binding partner of pRB protein in early 1990 [138]. RBP2 contains a highly conserved JmjC domain and was found as a specific H3K4me3/2 demethylase [30,139] (Figure 4). *Rbp2*^*−/−*^ mice are viable and display mild phenotypic defects in expansion of hematopoietic stem cells and myeloid progenitors. The weak phenotype of *Rbp2*^*−/−*^ mice suggests that other *JARID1* family proteins may compensate the loss of *Rbp2*[139].

During ES cell differentiation, RBP2 is dissociated from *HOX* genes, resulting in increased H3K4me3 levels and gene activation [30]. Consistently, Pasini et al. reported that RBP2 associates with the important Polycomb repressive complex 2 (PRC2), which enzymatically generates the repressive mark H3K27me3 for silencing of many differentiation-specific genes in ES cells [140]. A genome-wide chromatin immunoprecipitation (ChIP)-on-chip analysis revealed that RBP2 colocalizes on a subset of PRC2 target gene promoters in mouse ES cells. However, the interaction of RBP2 with PRC2 may not be strong, because the mass spectrometric analysis revealed that affinity eluates of the PRC2 component EED, which were purified from ES cell extracts, did not contain RBP2 [141]. Beshiri et al. recently demonstrated that RBP2 augments the repressive effects of the pRB-related protein p130 and E2F4 on cell cycle genes during stem cell differentiation via H3K4me3 demethylation [142]. Interestingly, RBP2 inhibits osteogenic differentiation of human adipose-derived stroma cells [143]. RBP2 interacts with Runt-related transcription factor 2 (RUNX2), a transcriptional factor that is required for osteogenic differentiation. Subsequently, RBP2 represses RUNX2 target genes, including *Alkaline phosphatase*, *Osteocalcin*, and *Osterix*[143].

#### JARID1B

JARID1B (PLU1 or KDM5B) was shown to be overexpressed in breast cancer cell lines [144]. As a member of the JARID1 family, PLU1 catalyzes the demethylation of H3K4me2/3. Its full activity requires JmjN, ARID, PHD_1_, and C_5_HC_2_ zinc finger in addition to the catalytic domain JmjC [30,34] (Figure 4). Consistent with the result of earlier studies, knockdown of PLU1 reduced MCF7 breast cancer cell proliferation and concomitantly upregulated expression of the *Breast cancer1, early onset* (*BRCA1*), *Caveolin 1* (*CAV1*), and *HOXA5* genes as a result of increased H3K4me3 levels on their promoters [34]. However, PLU1′s role in ES cell self-renewal and differentiation is controversial. Xie et al. reported that PLU1 is a downstream target of the pluripotent factor Nanog and is required for ES cell self-renewal [145]. PLU1 interacts with the chromodomain protein MRG15 and is recruited to H3K36me3-containing sites within gene bodies of self-renewal-associated genes via MRG15. Knockdown of PLU1 or MRG15 increased intragenic H3K4me3 that produces cryptic intragenic transcription and inhibited the transcriptional elongation [145]. Another study showed that constitutive overexpression of PLU1 blocked neural terminal differentiation [146]. On the contrary, Schmitz et al. has provided evidence that PLU1 is required for the neural differentiation of ES cells but is dispensable for self-renewal [147]. Using a genome-wide ChIP-sequencing analysis, they found that PLU1 predominantly localizes on the transcription start sites of target genes, over 50% of which are also occupied by Polycomb group proteins. PLU1-depleted ES cells fail to differentiate into the neural lineage, which correlates with the inappropriate depression of stem and germ cell genes [147]. These findings are further supported by their recent research in *Plu1* knockout mice, which have the phenotype of neonatal lethality and neural defects [148]. The discrepancies in these studies regarding the role of PLU1 in ES cell homeostasis are not entirely clear. However, Schmitz et al. indicated that their PLU1 localization data were obtained using a better PLU1 antibody and that the unimportance of PLU1 in ES cell self-renewal was confirmed by both a lentiviral shRNA knockdown method and a genetic deletion approach.

#### JARID1C and JARID1D

Compared with RBP2 and PLU1, much less is known about the biological function of JARID1C (SMCX or KDM5C) and JARID1D (SMCY or KDM5D). Both demethylases have similar domain structures and contain a conserved and functional JmjC domain that is responsible for demethylating H3K4me2/3 [30-32]. SMCX is an X-chromosome gene that escapes from X inactivation [149] and is often mutated in renal tumors and X-linked mental retardation (XLMR), suggesting that it has important functions in the human kidneys and brain [150,151]. Indeed, SMCX is highly expressed in brain during zebrafish development and is required for neuron survival [31]. Moreover, SMCX knockdown reduces dendritic length of rat primary neurons, which cannot be rescued by its XLMR-patient mutants with reduced demethylase activity [31]. Therefore, SMCX may play an important role in neuronal development. In addition, Outchkourov et al. reported that SMCX may interact with the transcriptional factors c-MYC and ELK1 to regulate gene expression in mouse ES cells [152].

JARID1D requires multiple domains, including ARID, JmjC, and C_5_HC_2_ zinc finger, for its full demethylase activity towards H3K4me3/2 [32] (Figure 4). JARID1D interacts with RING6A/MBLR, a polycomb-like protein with homology to Mel18 and Bmi1 proteins [153]. This interaction stimulates JARID1D’s enzyme activity *in vitro*; the protein complex mediates H3K4me3 demethylation at the *Engrailed 2* gene promoter and is required for *Engrailed 2* gene repression [32]. However, JARID1D’s biological role in stem cells is largely unknown. Given its localization on the Y-chromosome, it will be interesting to determine whether JARID1D plays a role in male-specific gene expression *in vivo*.

#### NO66

NO66 has been reported to demethylate H3K4me3/2/1 and H3K36me3/2 [124] and to catalyze histidyl hydroxylation of the 60S ribosomal protein Rpl8 [125]. This enzyme inhibits osteoblast differentiation [124]. Specifically, it directly interacts with Osterix, an osteoblast-specific transcription factor, and represses Osterix target gene expression [124]. In addition, NO66 plays a role in mouse ES cell differentiation [154]. During this process, it is recruited to stemness genes (e.g., *Oct4* and *Nanog*) via the PHD finger protein 19 (PHF19), which interacts with the H3K27 methyltransferase complex PRC2; NO66-PHF19-PRC2 represses gene expression by reducing H3K36me3 and increasing H3K27me3 [154].

## Conclusions

Stem cells are indistinguishable from somatic cells at the genomic level. In contrast, there are remarkable differences in epigenomes that may be represented by covalent and noncovalent modifications of histones and DNA. As reviewed herein, specific epigenetic modifiers, such as H3K4 methylation modifiers, may play fundamental roles in orchestrating cellular epigenomes whose genomic sequences are identical. Consistent with this, many H3K4 methylation modifiers and their components are required for ES cell self-renewal or differentiation. In addition, some of them cooperate with transcription factors for efficient somatic cell reprogramming. For example, WDR5 is required for the efficient generation of pluripotent stem cells that were induced by Oct4, Sox2, c-Myc, and Klf4 [47]. Therefore, the epigenetic modifiers, with the transcription factor network, may establish epigenomes in a coordinate manner.

Recently, small molecule inhibitors against specific histone methyltransferases, including LSD1 inhibitors, have been developed by several pharmaceutical companies, although their specificities and efficacies require improvement [155]. Certain inhibitors, alone or combined, may increase somatic reprogramming efficiency or drive somatic reprogramming, perhaps providing new avenues for personalized therapeutic interventions using stem cells. With regard to the roles of histone modifiers in stem cell maintenance and differentiation, many more new exciting findings are expected. We predict that our current and future knowledge about stem cell self-renewal and lineage commitment will be highly relevant to cancer stem cell studies, because stem cells and cancer stem cells share several characteristics, such as high degrees of self-renewal and differentiation [156]. We believe that a new era of stem cell epigenetics has begun.

## Competing interests

The authors declare that they have no competing interests.

## Authors’ contributions

BG prepared the initial draft of the paper. MGL initiated and modified the manuscript. Both authors read and approved the final manuscript.
